# The Effective Way of Botulinum Toxin Injection to Reduce Bite Force: Preliminary Study

**DOI:** 10.3390/toxins17100519

**Published:** 2025-10-21

**Authors:** Kun-Hwa Kang, Jae-Kwang Jung, Jin-Seok Byun, Ji Rak Kim

**Affiliations:** 1 Department of Oral Medicine, School of Dentistry, Kyungpook National University, Daegu 41940, Republic of Korea; 2 Craniofacial Nerve-Bone Network Research Center, Kyungpook National University, Daegu 41940, Republic of Korea

**Keywords:** bite force, botulinum toxin, masticatory muscles, medial pterygoid muscles

## Abstract

This study investigated bite force changes after botulinum toxin type A (BoNT-A) injection into different masticatory muscles. Thirty-five male participants were divided into three groups: masseter only (M), masseter and temporalis (MT), and masseter, temporalis, and medial pterygoid (MTP). Bite force was measured before and up to 6 months after injection with the Dental Prescale II system. Baseline values showed no significant group differences. Group M exhibited significant reduction at 1 and 2 weeks, with recovery within 1 month. Group MT showed a similar transient decrease, also recovering after 1 month. In contrast, Group MTP demonstrated a more pronounced and prolonged reduction, persisting up to 4 months before recovery. These results indicate that the extent and duration of BoNT-A effects depend on the number of muscles injected. Multi-muscle injections, including the medial pterygoid, provide more durable suppression. However, further research involving patient populations is needed to clarify whether multi-muscle injection strategies provide therapeutic benefits in clinical conditions such as temporomandibular disorders or oromandibular dystonia.

## 1. Introduction

Botulinum toxin type A (BoNT-A) injection has become increasingly used in the field of dentistry. It is used in the treatment of myofascial pain syndrome [1], tension-type headache [2], sleep bruxism [3], oromandibular dystonia [4], and recurrent dislocation of the temporomandibular joint [5], as well as an esthetic treatment to reduce muscular volume [6]. It is preferred by clinicians because of its clear effectiveness, minimal need for patient cooperation, and low risk of side effects. According to a systematic review on the safety and adverse effects of BoNT-A injection at masticatory muscles, the most common side effects were regional muscle weakness, tenderness at the injection site, and mild discomfort during chewing [7]. Additionally, these adverse effects were transient and resolved spontaneously [7].

The action mechanism of BoNT-A is the interfering of the release of acetylcholine-filled vesicles into the synaptic cleft, which causes a temporary functional denervation of neuromuscular end plates [8,9]. Muscular function gradually recovered with the collateral axonal sprouting [8]. Axonal sprouting after BoNT-A injection begins within the first 2 weeks, with visible unmyelinated sprouts emerging as early as 13 days post injection in mice [10]. These sprouts continue to grow in length and complexity over time, extending up to 47 days beyond as part of the recovery process [10]. As a result, the peripheral effect of BoNT-A reaches a maximum peak at 5–6 weeks following administration, depending on the type and size of the targeted muscles [11]. Neural signaling begins to normalize after 12 weeks, and functional recovery occurs between 3 and 6 months post-injection [8,12].

To complement these limitations, various studies have been conducted to enhance the efficacy and duration of BoNT-A injections. When different doses of 20, 30, and 40 units were injected into the masseter muscle, there were no statistically significant differences observed among the three dosage levels [13]. After administering a booster injection at 18 weeks following the initial injection, the significant effect was maintained up to 12 weeks, with the maximum reduction in voluntary clenching force observed at 6 weeks after the second injection [12]. Additionally, patients who received 2 to 8 repeated injections at a clinic over the course of a year showed a positive correlation between the number of injections and the average reduction in muscle size [14]. The author recommended 2 to 4 repeated injections per year to achieve the long-term effects of BoNT-A injections [14]. Although the prevalence is less than 5%, high dosage and long-term repeated injections are known to be major risk factors for BoNT-A resistance [15,16,17].

Additionally, because of repeated injections, a decrease of 6% in cortical thickness of the mandible was reported in humans, with no evident changes in either volume or density of bone. While in animals, significant bone loss of both cortical and trabecular compartments of the condylar region has been identified, but the underlying mechanisms remain unclear [18]. Therefore, research determining the optimal dosage and the most effective combination of injection sites to enhance the efficacy and duration of an initial injection has important implications.

Previous reports are mostly based on the injection of BoNT-A into the masseter muscle. Although the masseter muscle is a representative jaw-closing muscles, functioning alongside the temporalis and medial pterygoid muscles. Interestingly, all three muscles perform the function of jaw closure, but their origins and attachment sites are different. The direction of muscle movement occurs from the attachment site towards the origin. The masseter is generally composed of two layers [19]. The superficial part originates from the anterolateral region of the zygomatic arch and runs posteriorly to attach to the lateral inferior part of the mandibular ramus. The deep masseter originates from the posterior part of the inner surface of the arch and runs anteromedially [20]. The anterior part of the temporalis muscle originates from the posterior region of the orbital part of the zygomatic bone and the lateral region of the greater wing of the sphenoid bone, and it inserts into the anterior margin of the coronoid process. An adjacent bundle located on the anteromedial part of the temporalis originates from the infratemporal crest of the greater wing of the sphenoid bone and inserts into the anterior region of the medial surface of the coronoid process and the temporal crest [21,22]. The medial pterygoid muscle originated from the medial surface of the lateral pterygoid plate. The anteromedial part of the muscle was inserted into the postero-inferior area of the medial surface of the mandibular ramus and angle, and the posterolateral part was inserted into the superior region of the main muscle insertion [23,24]. All three muscles are innervated by branches of the mandibular nerve, a branch of the trigeminal nerve [23,25].

These muscles generate bite force by working together during the jaw closure but contributing unequally to the overall bite force [26]. The masseter muscle contributes approximately 43% of the intrinsic strength of the jaw-closing muscles, the temporalis muscle approximately 36% and the medial pterygoid approximately 21% [27]. Therefore, injecting BoNT-A into all three jaw-closing muscles may have a different effect on bite force reduction compared to injecting it into one or two of them.

However, there have been no studies reported comparing the effects of injections into each jaw-closing muscles on occlusal force. If the optimal injection sites and dosages are determined, it could lead to the establishment of a new treatment protocol for masticatory muscle disorders. This study aims to compare the differences in the reduction in occlusal force and the sustainable period among three groups based on injection sites: (1) masseter muscle, (2) masseter and temporalis muscles, and (3) masseter, temporalis, and medial pterygoid muscles.

## 2. Results

A total of 35 male patients (100%) were included in the study, with a mean age of 30.2 ± 8.6 years (range, 22–59 years). Participants were classified into three groups according to the injection sites of botulinum toxin: Group M (masseter only), Group MT (masseter and temporalis), and Group MTP (masseter, temporalis, and medial pterygoid) (Table 1).

### 2.1. Changes in Bite Force According to Injection Sites

#### 2.1.1. Masseter Group (M)

In the masseter group, the baseline mean maximum bite force was 868.9 ± 323.3 N. A significant reduction was observed at 1 week (718.3 ± 245.7 N, *p* = 0.0240) and 2 weeks (715.0 ± 320.9 N, *p* = 0.0232) after injection. At 1 week, bite force was reduced by −150.6 N (95% CI −271.0 to −30.2; Cohen’s d = 0.65), and at 2 weeks by −153.9 N (95% CI −285.2 to −22.6; d = 0.61), both representing moderate effect sizes. Thereafter, bite force gradually returned toward baseline. At 1 month, the mean reduction remained substantial (−151.5 N, 95% CI −340.2 to +37.2; d = 0.42), but the confidence interval included zero and the difference was not statistically significant after Bonferroni adjustment. No significant differences were observed from 1 month to 3 months (Table 2).

#### 2.1.2. Masseter and Temporalis Group (MT)

In the MT group, the baseline mean maximum bite force was 737.7 ± 309.9 N. A significant reduction was observed at 1 week (512.2 ± 233.4 N, adjusted *p* = 0.0012) and 2 weeks (424.8 ± 207.3 N, *p* = 0.0084) after injection. At 1 week, bite force was reduced by −225.5 N (95% CI −311.0 to −140.0; Cohen’s d = 1.89), and at 2 weeks by −312.9 N (95% CI −468.4 to −157.4; d = 1.44), both representing large effect sizes. Bite force subsequently recovered, with no significant differences from baseline between 1 and 4 months (Table 3).

#### 2.1.3. Masseter, Temporalis, and Medial Pterygoid Group (MTP)

In the MTP group, the baseline mean maximum bite force was 1000.4 ± 313.9 N. Bite force showed a pronounced reduction from 1 week (570.2 ± 261.1 N, *p* < 0.0001) through 4 months (714.2 ± 259.6 N, *p* = 0.0080), with the lowest value at 2 weeks (487.0 ± 263.6 N, *p* = 0.0008). At 1 week, bite force was reduced by −430.2 N (95% CI −636.7 to −223.7; Cohen’s d = 1.49), and at 2 weeks by −513.4 N (95% CI −720.7 to −306.1; d = 1.77), both representing very large effect sizes. At 1 month, the reduction remained substantial (−490.4 N, 95% CI −703.3 to −277.5; d = 1.65), and at 2 months the reduction was still significant (−415.5 N, 95% CI −622.3 to −208.7; d = 1.44). At 3 months, bite force was reduced by −361.0 N (95% CI −577.1 to −144.9; d = 1.20), indicating that the suppressive effect persisted for up to 4 months. No significant differences were observed at 5 months and 6 months, consistent with recovery toward baseline (Table 4).

### 2.2. Comparison Among Groups

**Figure 1:**
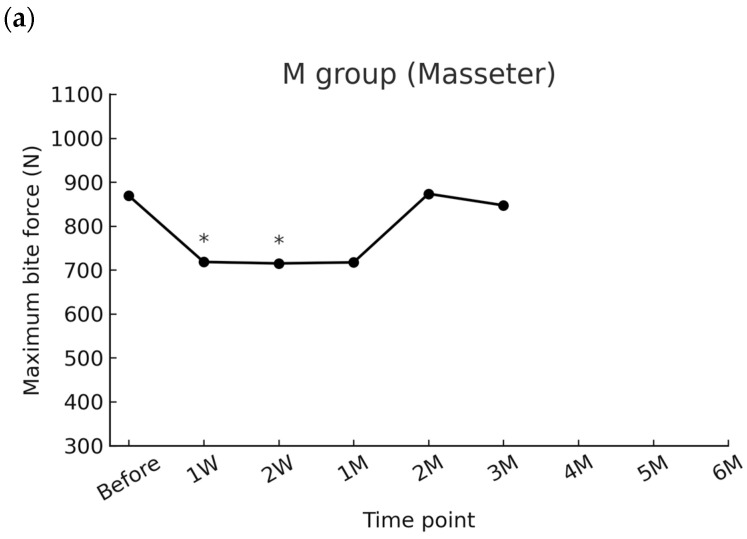

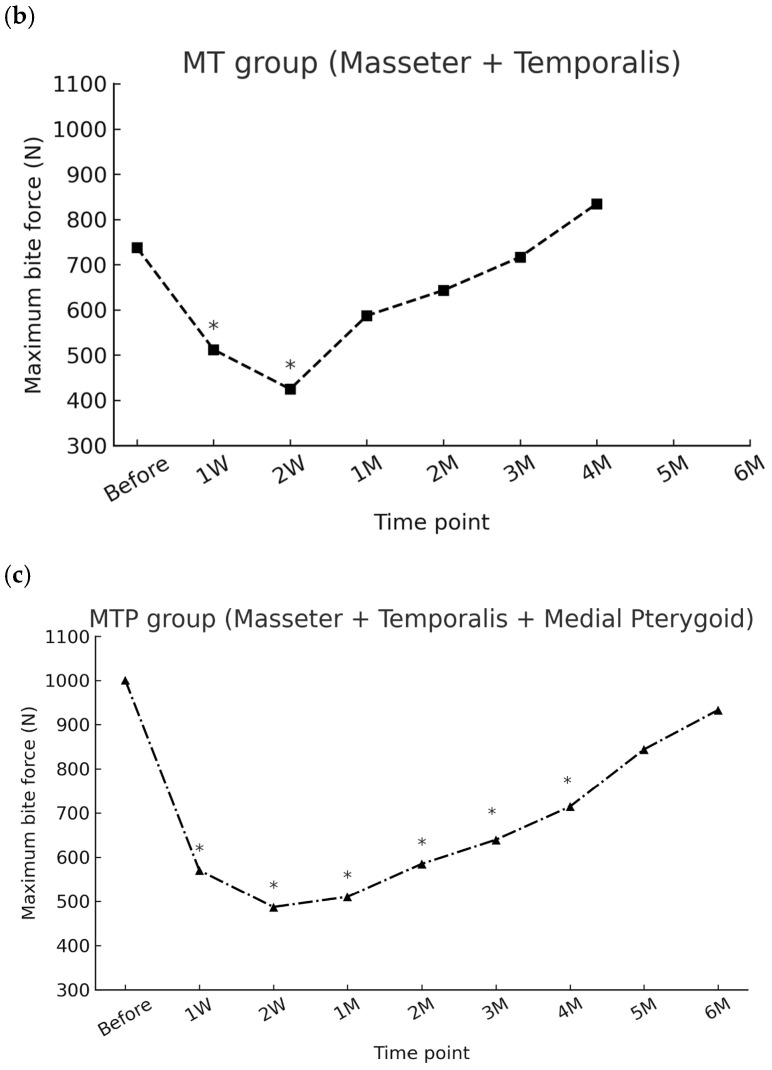
Time-course changes in maximum bite force across groups. Asterisks indicate significant differences compared with baseline after Bonferroni correction. (**a**) masseter group (**b**) masseter and temporalis group (**c**) masseter, temporalis, and medial pterygoid.

When comparing the three groups, no significant differences in baseline bite force were observed (*p* = 0.196). After injection, however, the reduction pattern differed across groups. In the M group, bite force significantly decreased at 1 week (718.3 ± 245.7 N, *p* = 0.0240) and 2 weeks (715.0 ± 320.9 N, *p* = 0.0232), but values recovered to baseline by 1 month (*p* = 0.1032). Similarly, in the MT group, significant reductions were noted at 1 week (512.2 ± 233.4 N, *p* = 0.0012) and 2 weeks (424.8 ± 207.3 N, *p* = 0.0084), followed by a return to baseline thereafter (all *p* > 0.05). In contrast, the MTP group demonstrated the most pronounced and sustained reduction, with bite force remaining significantly lower than baseline up to 4 months (all *p* < 0.01 through 4 M), before showing gradual recovery at 5 and 6 months (*p* = 0.1896 and *p* = 1.0, respectively). These results indicate that the extent and duration of bite force suppression were greatest in the MTP group (Figure 1, Appendix A Table A1).

**Figure 2:**
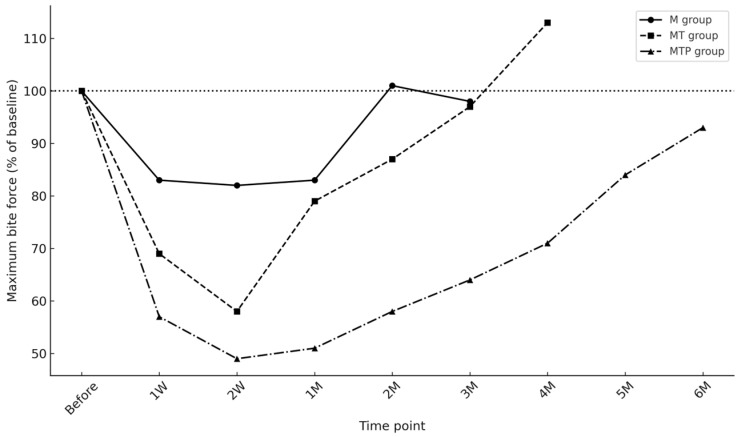
Relative changes in bite force compared with baseline.

When expressed as a percentage relative to baseline (set as 100%), the transient recovery of the M and MT groups became more evident, as both groups regained near-baseline levels by 1 month. Meanwhile, the MTP group demonstrated a >40% reduction at 2 weeks and did not reach full recovery until after 4 months, underscoring the prolonged suppressive effect of multi-muscle injections (Figure 2).

## 3. Discussion

Evaluation of the therapeutic effects of botulinum toxin can be performed using various methods, ranging from simple clinical observation of facial changes to advanced imaging techniques such as ultrasonography, 3-dimensional computed tomography, and magnetic resonance imaging, which allow assessment of muscle volume [14,28,29]. However, such volumetric changes represent a secondary consequence of muscle paralysis. For earlier and more direct evaluation of therapeutic efficacy, it is more appropriate to measure the reduction in muscular strength. In this regard, bite force measurement provides a practical and reliable means of assessing masticatory function.

Bite force measurements have been applied using different techniques. Early approaches involved stress–strain gauges placed between teeth, which allowed repeated measurements but had important limitations [30,31]. These gauges typically recorded force on a single tooth rather than across the dentition, and results were influenced by local occlusal conditions. In addition, the thickness of the sensors interfered with bite force accuracy, since maximum force is achieved in near-contact intercuspal positions. The Dental Prescale II system overcomes some of these limitations by using a thin (150 µm) film that exerts minimal influence on occlusion while also permitting comparisons across different occlusal conditions [32]. Nonetheless, careful handling is required to minimize operator error, and consumable film costs must be considered.

To reduce bias in this study, participants practiced biting paper sheets of similar thickness before actual measurements. Although repeated exertions can theoretically induce fatigue, each assessment lasted only ~3 s, with rest between trials, making substantial fatigue effects unlikely. Moreover, age and baseline bite force did not differ between groups, suggesting limited confounding from these variables. Body weight was not collected and remains a limitation. Individuals reporting subjective pain and those with suspected temporomandibular disorders were excluded, given the high sensitivity of bite force to pain and joint pathology. Despite these methodological precautions, bite force remains susceptible to variability, representing a limitation of the present study. Future investigations incorporating repeated measurements across several days may help to minimize intra-individual fluctuations and further validate bite force as an outcome measure in BoNT-A trials.

In cosmetic applications, botulinum toxin is generally injected only into the target muscle, most commonly the masseter, in order to reduce its volume in cases of masseter hypertrophy. However, when the therapeutic goal is to reduce parafunctional activities such as nocturnal bruxism or abnormal mandibular movements, it is necessary to diminish the functional output of the masticatory system as a whole rather than a single muscle [33,34].

There are four principal muscles of mastication: the masseter, temporalis, medial pterygoid, and lateral pterygoid. Among these, the lateral pterygoid primarily contributes to mandibular opening, whereas the other three muscles (masseter, temporalis, and medial pterygoid) are mainly responsible for mouth closing and food mastication. Bite force is generated by the integrated activity of these three muscles, and thus effective reduction in bite force requires consideration of all three rather than the masseter alone [35,36]. A possible clinical phenomenon following botulinum toxin injection is compensatory hyperactivity of adjacent muscles, sometimes referred to as the Whac-A-Mole phenomenon [37,38]. When one muscle is weakened, other muscles may potentially become hyperactive, which could lead to fatigue or discomfort. Clinically, some patients receiving injections limited to the masseter have been reported to experience temporalis discomfort within one to two weeks, suggesting possible compensatory recruitment. Moreover, previously weakened muscles may later regain activity as part of such compensatory mechanisms.

Therefore, in order to effectively reduce overall bite force and prevent compensatory hyperactivity, it may be more appropriate to target not only the masseter but also the temporalis and medial pterygoid muscles. This strategy is consistent with the pharmacological mechanism of BoNT-A, which blocks acetylcholine release at the neuromuscular junction and modulates abnormal muscle activity [39,40]. This strategy provides a more balanced reduction in masticatory function and may help achieve sustained therapeutic benefits in patients with bruxism. The present study directly compared outcomes among patients receiving injections into the masseter alone, the masseter and temporalis, or all three muscles, thereby providing clinical evidence to support optimal treatment strategies. No participants reported adverse effects such as injection-site pain, infection, or persistent muscle weakness following BoNT-A administration in the three masticatory muscles. A few individuals experienced transient difficulty in chewing due to reduced bite force; however, these symptoms resolved spontaneously without additional intervention during follow-up. Importantly, no participants were withdrawn because of adverse effects, and loss to follow-up in the MT and MTP groups was attributable solely to missed hospital visits rather than treatment-related complications.

The present study demonstrates that the extent and duration of bite force reduction following BoNT-A injection are closely related to the number of masticatory muscles treated. While injections limited to the masseter or to the masseter and temporalis muscles resulted in only transient reductions, with recovery within 1 month, the MTP group produced a more sustained suppressive effect lasting up to 4 months. From a clinical perspective, these findings suggest that the therapeutic target and injection strategy should be carefully considered depending on the treatment objective. Since bite force is closely linked to overall masticatory performance, reductions observed in this study may have broader functional implications in patients with parafunctional habits [41,42]. For conditions such as masseteric hypertrophy or mild temporomandibular disorder, limiting injections to the masseter or masseter and temporalis may be sufficient, as the effect is transient and recovery occurs relatively quickly. However, in cases where a more durable reduction in bite force is desirable—for example, in patients with severe bruxism or high occlusal loading contributing to temporomandibular joint degeneration—multi-muscle injections, including the medial pterygoid, may provide a longer-lasting therapeutic benefit. Nevertheless, it should be noted that the present findings are derived exclusively from healthy participants, and the responses of patients with bruxism, myofascial pain, or temporomandibular disorders may differ. This limitation restricts the direct clinical generalizability of the results. However, establishing the pattern of bite force reduction according to different muscle combinations in healthy individuals is a necessary preliminary step before applying such protocols to patient populations. In this regard, the present study is intended not to generalize directly to clinical practice but to suggest future research directions and demonstrate potential feasibility. Future controlled studies including both healthy individuals and patient populations are warranted to determine whether multi-muscle injections yield comparable or more pronounced therapeutic benefits in clinical settings.

Another important consideration is whether treatment efficacy should be enhanced by increasing the dosage or by distributing the same dose across multiple muscles. In the present study, 50 units of BoNT-A were administered in the M group, whereas 100 units were used in both the MT and MTP groups. The greater reduction in bite force observed in the latter groups is partly attributable to the higher overall dose, which is expected. However, our findings indicate that distributing the same total dose across multiple muscles, rather than concentrating it in a single site, leads not only to greater suppression of bite force but also to a longer duration of effect. Specifically, dividing the dose to include the medial pterygoid muscle yielded both a larger reduction in bite force and a slower recovery, highlighting the importance of injection distribution in clinical outcomes. Previous studies have reported no significant difference in efficacy between 50 and 70 units administered to the masseter, suggesting that increasing the dose beyond 50 units in a single muscle yields limited additional benefit [43]. Moreover, because BoNT-A is commercially available in vials containing either 50 or 100 units, the practical dosage options were inevitably constrained. Consequently, when additional muscles beyond the masseter were included, a total dose of 100 units was employed. The primary objective of this study was therefore to explore how this fixed total dose could be most effectively distributed among different masticatory muscles. The present findings provide preliminary evidence that inclusion of the medial pterygoid may enhance therapeutic outcomes, and further research is warranted to determine the optimal allocation of a 100-unit dose across the three muscles to maximize clinical efficacy.

It must also be emphasized that the principal strength and limitation of BoNT-A therapy lies in its reversibility. While this transient nature is advantageous for safety, it also necessitates repeated injections or booster shots to maintain long-term efficacy in uncontrolled bruxism or abnormal mandibular movement disorders. Frequent reinjection, however, raises concerns of antibody-mediated resistance and diminished therapeutic effects [44]. Therefore, strategies that maximize the duration of each treatment cycle may help reduce the need for frequent reinjections and mitigate the risk of secondary non-responsiveness [45,46]. In this regard, administering smaller doses across multiple masticatory muscles, rather than repeatedly injecting a single muscle, may help to minimize the risk of antibody formation and resistance while providing greater overall benefit to patients. Taken together, the present results provide evidence-based guidance for tailoring BoNT-A injection strategies in the management of masticatory muscle hyperactivity and related disorders, highlighting the importance of both injection site selection and dose distribution in determining the magnitude and duration of therapeutic effect.

The present study has several limitations. First, the relatively small sample size may restrict the generalizability of the findings. Recruitment was challenging because only healthy individuals without temporomandibular disorder symptoms were eligible, resulting in about 10 participants per group, with slightly more in the masseter-only group due to the cosmetic benefit of masseter injection. These findings should therefore be interpreted as exploratory evidence rather than definitive conclusions, and future large-scale studies are warranted to validate these results. Second, only male participants were included, and thus potential sex-related differences in bite force reduction after BoNT-A injection were not addressed. Male and female may exhibit differences in certain aspects of feeding and mastication behaviors. Gender differences in masticatory performance and in the spatial-temporal parameters of masticatory movements, including the path and rhythm, have been observed [47,48]. These gender differences may serve as a variable that could affect the duration of the effect after BoNT-A injection. Although both sexes were initially intended, no female participants were enrolled, and the study was therefore limited to men. This restricts generalizability, and future studies should include both sexes to clarify potential sex-related differences in BoNT-A effects. Third, the lateral pterygoid muscle was not included in the injection protocol. Although its primary function is mouth opening, the lateral pterygoid also contributes to lateral mandibular movements and plays a role in the final phase of clenching; therefore, its involvement may influence bite force outcomes. Fourth, this study was not designed with a control group, and neither the intervention nor the follow-up assessments were blinded. Because the study involved healthy volunteers, obtaining consent for multiple saline placebo injections was considered impractical, and we judged the placebo effect to be minimal given the well-established mechanism of BoNT-A. Nonetheless, the absence of blinding and a placebo arm may have introduced bias and limited the internal validity of the results. Future studies should adopt blind designs with appropriate control groups to provide more objective evidence. Fifth, subjective patient assessment scales for the improvement of symptoms after the injection were not evaluated. Future investigations should include larger, more diverse populations and incorporate injections into the lateral pterygoid muscle to provide a more comprehensive understanding of masticatory muscle function. Longitudinal trials assessing repeated or booster injections will also be valuable for determining optimal treatment intervals and minimizing the risk of BoNT-A resistance.

## 4. Conclusions

BoNT-A injections effectively reduce bite force, with the extent and duration of reduction determined by the number of masticatory muscles treated. Multi-muscle injection strategies, particularly those including the medial pterygoid, may provide a longer-lasting therapeutic effect compared with single-muscle approaches.

## 5. Materials and Methods

### 5.1. Participants

A total of 35 healthy adult participants were recruited from patients who visited Kyungpook National University Dental Hospital. Because this study focused exclusively on healthy individuals, no specific inclusion criteria were applied beyond the absence of temporomandibular disorders or other conditions that could affect bite force. Participants with abnormal occlusal conditions that could affect bite force, such as missing teeth, open bite or cross bite, were excluded based on clinical assessment [49]. Additionally, patients with a history of oral and maxillofacial surgery, serious systemic disease, current use of medications affecting muscle relaxation, contraindications to BoNT-A, and previous or current BoNT-A injection within 6 months of starting the study were also excluded [50].

To minimize confounding factors influencing bite force, only individuals without a diagnosis of temporomandibular disorders, as confirmed by the Diagnostic Criteria for Temporomandibular Disorders (DC/TMD) examination, were included. Participants with myofascial pain, headache, or other pain-related conditions were excluded. Furthermore, in relation to bruxism, only individuals who reported no oral parafunctional habits on the screening questionnaire were enrolled.

This research was reviewed and approved by the Institutional Review Board of the Kyungpook National University Dental Hospital (KNUDH-2023-03-03-01). Informed consent was obtained from each participant according to the ethical guidelines.

**Figure 3:**
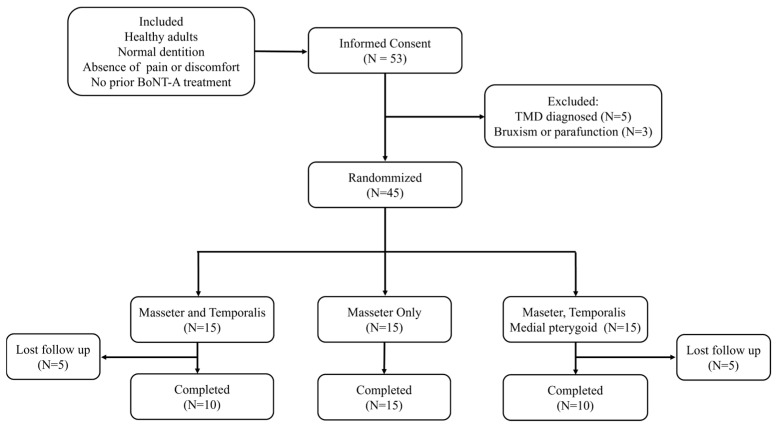
Flowchart of participant selection and study completion.

The total study period lasted 12 months. Each participant received the first injection at different time points, and all participants completed their first injection within 6 months. Participant selection and dropout rates are presented in a flow diagram (Figure 3).

### 5.2. Intervention

The injection material was BoNT-A (WonderTox 100 units, Chong Kun Dang, Seoul, Republic of Korea), prepared by diluting 100 mouse units (U) in 2 mL of normal saline according to the manufacturer’s instructions. After providing detailed information about the study protocol, written informed consent was obtained from all participants. Subsequently, participants were assigned to one of the three treatment groups in the order of enrollment. Each group received injections into different combinations of masticatory muscles as described below.

**Figure 4:**
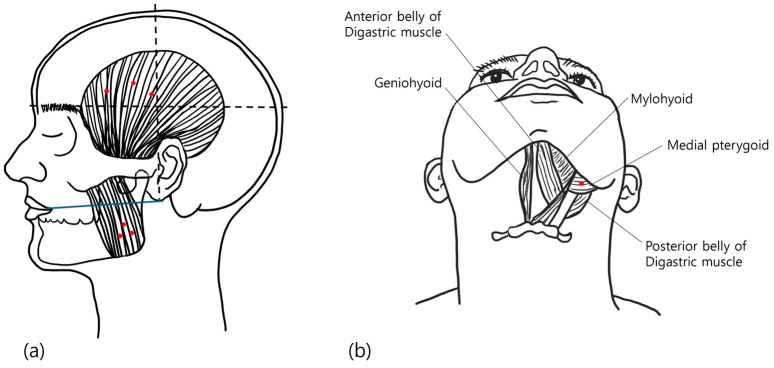
Diagram of the Injection sites. (**a**). The upper border of the masseter insertion site is a line connecting the inferior border of the ear lobe and the corner of the mouth, and the lower boundary is the inferior ridge of the mandible. The anterior and posterior borders are determined through the muscle palpation. The lower boundary of the temporalis insertion site is a line above the eyebrow, parallel to the zygomatic arch, and the posterior boundary is a vertical line passing through the anterior border of the ear. The insertion points were determined within the internal areas of the boundaries, avoiding visible blood vessels and through palpation. (**b**). The medial pterygoid muscle was injected via the submandibular route. with the patient lying supine position and lifting their chin, the needle was inserted medially to the mandibular angle and advanced along the inside of the mandible to a depth of 10–15 mm. The actual injection points are marked in red.

Group M was administered 25 U BoNT-A at three points in each masseter muscle (total dose 50 U) [38]. The anterior border and most prominent bulge of the masseter muscle was identified during jaw clenching, with injections inferior to a line connecting the inferior border of the ear lobe and angle of the mouth [38,51]. Group MT was administered 25 U in each masseter muscle and 25 U at three sites in each temporalis muscle (total dose 100 U), identified by palpation during jaw clenching [38,51]. Group MTP was administered 25 U in each masseter muscle, 15 U in each temporalis muscle and 10 U into a single site in the medial pterygoid muscles bilaterally (total dose 100 U) [38]. The medial pterygoid muscle was injected extraorally via a submandibular route with the patient lying supine with the neck extended. The needle was inserted medially to the mandibular angle and progressed parallel to the inside of the mandible to a depth of 10–15 mm [38,50,51,52] (Figure 4). A 29 gauge needle was used at all injection sites.

### 5.3. Bite Force Measurement

To measure occlusal load, a system consisting of pressure-sensitive sheets (Dental Prescale II, Fuji Film, Tokyo, Japan) and an analyzing device (Bite Force Analyzer, GC corporation, Tokyo, Japan) was used. The Dental prescale II device can measure the contact area (mm^2^), mean occlusal pressure (MPa), and integrated occlusal load (N). To evaluate changes in the contact area, a contact area analysis device (Bite Eye, GC Dentalcorporation, Tokyo, Japan) is used.

Each subject was seated in a relaxed upright position with the Frankfort horizontal plane and instructed to bite the test sheet as hard as possible for 3 s [29]. The occlusal load was scanned and calculated by the computerized system. To prevent measurement errors, practice trials were conducted before the evaluation, and measurements were taken three times with rest intervals. Given the short duration and recovery time, fatigue effects were considered negligible. The average of the three trials was used as the final value. Occlusal load was measured before the injection and at 1 week, 2 weeks, 4 weeks, 2 months, 3 months, 4 months, 5 months, and 6 months after the injection. The differences in overall bite force (N) before and after the injection were compared. All injections and recordings were performed by the same investigator (J.R.K.). For each group, follow-up measurements were discontinued once bite force had returned to baseline values.

### 5.4. Statistical Analysis

All statistical analyses were performed using IBM SPSS Statistics for Windows, version 27.0 (IBM Corp., Armonk, NY, USA). Data distribution was assessed with the Shapiro–Wilk test. Baseline differences among the three groups were analyzed using one-way ANOVA. Paired t-tests were performed to compare baseline (before injection) with each subsequent time point. To account for multiple comparisons, Bonferroni correction was applied based on the number of time point comparisons within each group (i.e., m = 5, 6, or 8). Statistical significance was assessed by comparing unadjusted *p*-values to the corresponding Bonferroni-adjusted alpha levels (α/m), all of which were less than 0.05. Adjusted *p*-values are reported throughout. This correction was applied only to within-group comparisons across repeated time points.

## Figures and Tables

**Table 1:** Baseline characteristics of patients according to botulinum toxin injection sites.

Variables	M (n = 15)	MT (n = 10)	MTP (n = 10)	*p*-Value
Age (years)	28.9 ± 9.7	30.2 ± 8.9	32.2 ± 6.9	0.661
Baseline bite force (N)	868.9 ± 323.3	737.7 ± 309.9	1000.4 ± 313.9	0.196

M, Masseter only; MT, masseter and temporalis; MTP, Masseter, temporalis, and medial pterygoid; Values are given as mean ± SD. *p*-value was obtained from ANOVA test.

**Table 2:** Changes in maximum bite force at each time point in the masseter (M) group.

Time Point	Mean (N)	SD (N)	Raw *p*-Value *	Adjusted *p*-Value **
Before	868.9	323.3	Reference	Reference
1 week	718.3	245.7	0.004	0.024
2 weeks	715.0	320.9	0.003	0.023
1 month	717.4	290.0	0.017	0.103
2 months	873.5	269.6	0.093	0.620
3 months	847.2	285.5	0.500	1.000

* Raw *p*-values were obtained from paired *t*-tests comparing baseline with each time point. ** Adjusted *p*-values were calculated using Bonferroni correction to account for five comparisons (m = 5). Statistically significant adjusted *p*-values (*p* < 0.05) are highlighted in bold.

**Table 3:** Changes in maximum bite force at each time point in the masseter and temporalis (MT) group.

Time Point	Mean (N)	SD (N)	Raw *p*-Value *	Adjusted *p*-Value **
Before	737.7	309.9	Reference	Reference
1 week	512.2	233.4	0.0002	0.0013
2 weeks	424.8	207.3	0.0014	0.0083
1 month	587.1	281.7	0.0708	0.4248
2 months	643.3	365.9	0.7063	1.0000
3 months	716.8	325.8	0.8490	1.0000
4 months	834.5	284.9	0.1523	1.0000

* Raw *p*-values were obtained from paired *t*-tests comparing baseline with each time point. ** Adjusted *p*-values were calculated using Bonferroni correction to account for six comparisons (m = 6). Statistically significant adjusted *p*-values (*p* < 0.05) are highlighted in bold.

**Table 4:** Changes in maximum bite force at each time point in the masseter, temporalis, and medial pterygoid (MTP) group.

Time Point	Mean (N)	SD (N)	Raw *p*-Value *	Adjusted *p*-Value **
Before	1000.4	313.9	Reference	Reference
1 week	570.2	261.1	<0.0001	<0.0001
2 weeks	487.0	263.6	0.0001	0.0008
1 month	510.0	280.5	0.0002	0.0016
2 months	584.9	262.0	0.0002	0.0016
3 months	639.4	289.8	0.0006	0.0032
4 months	714.2	259.6	0.0010	0.0080
5 months	844.0	347.1	0.0180	0.1896
6 months	932.9	253.5	0.5283	1.0000

* Raw *p*-values were obtained from paired *t*-tests comparing baseline with each time point. ** Adjusted *p*-values were calculated using Bonferroni correction to account for eight comparisons (m = 8). Statistically significant adjusted *p*-values (*p* < 0.05) are highlighted in bold.

## Data Availability

The original contributions presented in this study are included in the article. Further inquiries can be directed to the corresponding author.

## Abbreviations

The following abbreviations are used in this manuscript:

BoNT-A	Botulinum Toxin Type A
M	Masseter
MT	Masseter and temporalis
MTP	Masseter, temporalis, and medial pterygoid
U	Unit

## Appendix A

**Table A1:** Mean differences, 95% confidence intervals (CI), and Cohen’s d effect sizes for bite force reduction relative to baseline across groups.

Group	Time Point	Mean Difference (N)	95% CI	Cohen’s d
M group	1 week	−150.6	−271.0 to −30.2	0.65
	2 weeks	−153.9	−285.2 to −22.6	0.61
	1 months	−151.5	−340.2 to +37.2	0.42
	2 months	4.6	−157.8 to +166.9	0.02
	3 months	−21.7	−211.4 to +168.0	0.07
MT group	1 week	−225.5	−311.0 to −140.0	1.89
	2 weeks	−312.9	−468.4 to −157.4	1.44
	1 months	−150.6	−316.9 to +15.7	0.65
	2 months	−32	−225.1 to +161.0	0.14
	3 months	−20.9	−210.5 to +168.7	0.09
	4 months	96.8	−112.3 to +305.9	0.3
MTP group	1 week	−430.2	−636.7 to −223.7	1.49
	2 weeks	−513.4	−720.7 to −306.1	1.77
	1 months	−490.4	−703.3 to −277.5	1.65
	2 months	−415.5	−622.3 to −208.7	1.44
	3 months	−361	−577.1 to −144.9	1.2
	4 months	−286.2	−492.0 to −80.4	0.95
	5 months	−156.4	−386.7 to +73.9	0.45
	6 months	−67.5	−288.4 to +153.4	0.21

Negative values indicate reductions relative to baseline; Cohen’s d represents absolute effect size.
